# DAAs in pregnancy are the only potential intervention to decrease vertical transmission: Pregnancy DAA data support safety

**DOI:** 10.1097/HC9.0000000000000924

**Published:** 2026-04-13

**Authors:** Catherine Chappell, Mia J. Biondi, Tatyana Kushner

**Affiliations:** 1 Department of Obstetrics, Gynecology, and Reproductive Sciences, University of Pittsburgh, Pittsburgh, Pennsylvania, USA; 2 School of Nursing, York University, Toronto, Ontario, Canada; 3 Viral Hepatitis Care Network, University Health Network, Toronto, Ontario, Canada; 4 Department of Gastroenterology and Hepatology, Weill Cornell Medicine, New York, New York, USA

## URGENCY OF HEPATITIS C TREATMENT IN PREGNANCY

Offering direct-acting antivirals (DAAs) in pregnancy is currently recommended by the American Association for the Study of Liver Diseases/Infectious Disease Society of America (AASLD/IDSA) as a part of decision-making, with multiple clinical and observational studies presented/published or underway. Treatment in pregnancy has major benefits. Pregnancy may be the only time when a person with hepatitis C virus (HCV) is engaged in care, and therefore the only opportunity to receive curative therapy. While curing HCV has the overall benefit of preventing onward community transmission and advanced liver disease, there are additional benefits in the prenatal population. Treatment can improve maternal outcomes and, importantly, prevent vertical transmission.^1^^–^^3^ The most recent meta-analysis evaluating rates of vertical transmission showed higher rates than previously cited.^4^^,^^5^ Unfortunately, vertical transmission remains the predominant reason for pediatric HCV worldwide, and pediatric cohort studies have shown that even after curative DAA therapy, children who acquired HCV vertically can develop fibrosis and cirrhosis.^6^

## INTERPRETATION OF DATA ON DAA EXPOSURE IN PREGNANCY

To prevent vertical transmission at a population level by treating HCV in pregnancy, it will be important to ensure knowledge translation of clinical data to the hepatology community, and in parallel, obstetrical and primary care providers. Careful consideration is required when interpreting maternal and fetal outcomes evaluating DAA safety in pregnancy, and must be compared with baseline outcomes in both the general perinatal population and among those with HCV in pregnancy, which have a higher rate of poor pregnancy outcomes due to comorbidities such as substance use disorder. This is important for the interpretation of current data because shared decision-making means to at least offer and discuss treatment as an option, which may not occur if there is a misinterpretation of the safety data by providers.

## RECENTLY REPORTED DATA FROM PHARMACOVIGILANCE DATASET AND INTERPRETATION OF FINDINGS

Recently, the largest dataset available on pregnancy outcomes with sofosbuvir/velpatasvir (SOF/VEL) exposure in pregnancy was presented at The Liver Meeting 2025. (Table 1).^7^ In total, 123/335 had pregnancy outcomes: 74/123 (60.2%) were live births, 28 (22.8%) were spontaneous abortions, and 21 (17.1%) were induced abortions. These outcomes are in line with the reported spontaneous abortion rate was similar to the rate in the United States of ~20% among all pregnancies;^8^ and importantly, 9 (32%) reported other conditions that contributed to the spontaneous abortion (recurrent pregnancy loss, substance use, other medication exposures, maternal hepatic failure).

**TABLE 1:** Reported congenital anomalies among live births and the trimester of DAA exposure^1^

Country	Data source	Trimester of SOF/VEL initiation	Congenital anomaly reported
Brazil	Spontaneous	Unknown	Kidney malformation
Canada	Study IN-CA-337-2100	First	Fallot’s tetralogy; congenital ureteric anomaly; congenital hydronephrosis
United States	Spontaneous	First	Fetal growth restriction; limb asymmetry
United States	Study IN-US-342-5634	Second	Talipes
United States	Study IN-US-342-5634	Second	Cryptorchism
United States	STORC study	Third	Congenital megacolon
United States	STORC study	Second	Retrognathia
United States	STORC study	Third	Pyloric stenosis
United States	STORC study	Third	Periauricular skin tag

Abbreviations: DAA, direct-acting antiviral; SOF, sofosbuvir; VEL, velpatasvir.

When evaluating the data, gestational age during DAA exposure matters, as recent observational and clinical studies have started DAA therapy in the second or third trimester, whereas early exposure is less well understood. During the first 4 weeks of gestation, the embryo is undifferentiated, and exposure to a toxic agent could impact fetal loss; however, the critical developmental window is 4–10 weeks of gestation, where a drug exposure may or may not be related to a birth defect.^9^ Understanding the pathogenesis of fetal anomalies is crucial when evaluating medication safety data.

Of the reported outcomes, 10 congenital anomalies were reported, 8.1% of the 123 pregnancies, 9 among the live births, and 1 among the spontaneous abortions, 6 of which occurred with second and/or third trimester exposure. Of the abnormalities reported following DAA exposure in the first trimester (limb asymmetry and Tetralogy of Fallot), there was no unifying anomaly associated with early drug exposure. Both limb asymmetry and the cardiac anomaly Tetralogy of Fallot are very different anomalies and unlikely to be caused by the same mechanism. Likewise, among the 6 fetal anomalies reported during prospective studies where SOF/VEL was initiated in pregnancy in the second and third trimester,^1^^,^^2^ it is biologically impossible for those anomalies to be due to SOF/VEL because the exposure occurred after organogenesis, thus the anomalies were present before initiation of SOF/VEL. In general, higher rates of adverse events are observed in clinical studies as compared with real-world studies, as a result of close participant monitoring. A salient example of this phenomenon in pregnancy is the antiretroviral pregnancy registry, which reported a 3–4 times higher rate of congenital anomalies, and when the anomalies were restricted to the first 7 days of life, they returned to a background rate of 2.4%.^10^

While additional studies are underway of DAA use in pregnancy, the data to date, including in the pharmacovigilance study of SOF/VEL, have demonstrated no major safety signals in the context of first-, second-, or third-trimester exposure. Appropriate interpretation of registry reports hinges on understanding baseline prevalence of adverse pregnancy outcomes. Specifically, the timing of gestational age exposure to SOF/VEL, the prospective data sources, and the background rate of spontaneous abortion help to contextualize the findings.

## LEVERAGING EXISTING DATA TO GUIDE DECISION MAKING ON TREATMENT DURING PREGNANCY

The interpretation of pregnancy outcomes data is complicated and nuanced, but shared decision making in deciding on HCV treatment during pregnancy is especially important considering multiple studies demonstrating the acceptability of HCV treatment in pregnancy,^11^^,^^12^ and the major benefit of prevention of vertical transmission.^1^^–^^3^ Thus, if a patient becomes pregnant while on SOF/VEL, while treatment could be stopped, the critical window of exposure has already occurred at the point of a positive pregnancy test. Therefore, stopping treatment may not mitigate potential harm. On the other hand, stopping treatment in this context could actually reduce the likelihood of achieving SVR, while also increasing the risk of maternal complications related to HCV and perinatal transmission. In the context of second or third trimester exposure, this later start likely mitigates any risk of fetal anomalies or spontaneous abortion caused by SOF/VEL, and our data to date supports the safety of HCV treatment started after 20 weeks of gestation.^1^^,^^2^ Treatment during pregnancy should be considered: this intervention is needed to prevent vertical transmission and work toward the WHO goal of hepatitis C elimination by 2030, which is only a few years away.
